# Reliability of ADR Jumping Photocell: Comparison of Beam Cut at Forefoot and Midfoot

**DOI:** 10.3390/ijerph20115935

**Published:** 2023-05-24

**Authors:** Jose M. Jimenez-Olmedo, Alfonso Penichet-Tomas, Basilio Pueo, Lamberto Villalon-Gasch

**Affiliations:** Research Group in Health, Physical Activity, and Sports Technology (Health-Tech), Faculty of Education, University of Alicante, 03690 San Vicente del Raspeig, Spainbasilio@ua.es (B.P.); lamberto.villalon@ua.es (L.V.-G.)

**Keywords:** accuracy, consistency, sensibility, countermovement jump, error, between-methods

## Abstract

The ability to detect small changes in a vertical jump is crucial when data are used by sports science specialists to monitor their athletes. This study aimed to analyze the intrasession reliability of the ADR jumping photocell and the reliability relative to the position of the transmitter when it is located facing the phalanges of the foot (forefoot) or the metatarsal area (midfoot). A total of 12 female volleyball players performed 240 countermovement jumps (CMJ), alternating both methods. The intersession reliability was higher for the forefoot method (ICC = 0.96; CCC = 0.95; SEM = 1.15 cm; CV = 4.11%) than for the midfoot method (ICC = 0.85; CCC = 0.81; SEM = 3.68 cm; CV = 8.75%). Similarly, the sensitivity values were better for the forefoot method (SWC = 0.32) than for the midfoot method (SWC = 1.04). Significant differences were found between the methods (13.5 cm, *p* < 0.05, ES = 2.1) with low agreement (*r_s_* = 0.57; ICC = 0.49; CCC = 0.15; SEM = 4.7 cm) and heteroscedasticity was observed (*r^2^* > 0.1). In conclusion, the ADR jumping photocell is shown to be a reliable tool for measuring CMJs. However, the reliability of the instrument can be influenced depending on the placement of the device. Comparing the two methods, the midfoot placement was less reliable as indicated by higher values of SEM and systematic error, and thus its use is not recommended.

## 1. Introduction

Measuring vertical jump height (VJH) is an important tool for evaluating lower body strength and power in athletes [1]. Lower body strength and power are critical components of athletic performance, as they enable activities such as jumping, sprinting, and changing direction quickly. The ability to generate force rapidly and explosively is especially important in sports that involve jumping or quick bursts of movement. Thus, the vertical jump test is widely used as a reliable and valid measure of lower body strength and power in athletes. It is an important tool for coaches and trainers to assess the functional capacity of their athletes and to track progress and fatigue over time [2,3,4,5]. Additionally, the vertical jump test may be used as a predictor of success in certain sports, as a higher vertical jump is often associated with better performance. A necessary condition to obtain precise values of the measurement of vertical jump is reliability. The consistency and ability to detect small changes in the devices used by specialists in sports sciences is a critical factor when collecting data, with the aim of controlling, evaluating, analyzing, and planning the prescription of physical exercise [6,7].

Motion capture systems and force plates are considered the gold standard in VJ estimation due to their high values of validity and reliability [8,9]. However, the use of both technologies is usually limited to research or high-performance centers due to their high cost. Because of these high prices and given the demand by specialists to have tools that allow them to determine the vertical jump, numerous instruments have proliferated, which are more manageable and cheaper and which allow estimating the vertical jump in all types of populations and situations [10]. All these new instruments, such as accelerometers, jump mats, or tailored video-based systems are more affordable alternatives than force platforms and motion capture systems. In addition, they exhibit higher ecological validity, as they can be administered in real-world conditions or be closely simulated in testing [11,12].

Photocells are part of these new devices capable of estimating jump height. They measure flight time by detecting the landing and take-off instants using an infrared (IR) beam [1]. These were originally designed to calculate velocity in linear sprints [13], but, in recent years, new devices designed more specifically for measuring vertical jumps have emerged, proving to be valid tools in VJH estimation [14,15]. The main advantage of these types of photocells compared to jump mats, force platforms, or photogrammetry is that they allow data collection to be carried out on a multitude of surfaces, being able to imitate the real conditions of a certain activity, obtaining the data instantly [16]. In addition, photocells have demonstrated high test-retest reliability. In this sense, the Fitjump device (Sports, Afyonkarahisar, Turkey) presented high consistency rates in the test-retest between sessions (ICC = 0.97; CV = 2.3%; SEE = 1.3 cm) [17]. Along the same lines, the Optojump photocell (Optojump, Microgate, Bolzano, Italy) obtained very high reliability values in the test-retest (ICC = 0.98; CV = 2.7%; SEE = 2.81 cm) [15]. Similar results were found by Rago et al. (2018) [8] using Optojump, although with degrees of reliability somewhat lower but equally high (ICC = 0.87; CV = 4.2%). Finally, the test-retest reliability of the ADR jumping device (ADR, Toledo, Spain) is also high (ICC = 0.89) [18]. On the other hand, photocells also show low sensitivity values: smallest detectable change (SDC) of 1.2 cm for Optogait, 1.1 cm for ADR, and the smallest worthwhile change (SWC) of 1.41 cm for wheeler jump (Wheeler Sports Tech, Tampa, FL, USA) [19].

However, these tests were carried out in different sessions (between sessions reliability), usually separated by a week. This period can generate changes in the original conditions of the sample that could influence the device reliability values. No studies have analyzed the intrasession reliability of these instruments. Additionally, despite the manufacturer’s claim of the system’s reliability for any foot position, as long as the forefoot intersects with the beam during landing, it is uncertain whether the system’s reliability is influenced by variable foot positioning during both take-off and landing in relation to the beam. This ambiguity arises due to the tendency of flight-time-based instruments to overestimate jump height, which can be attributed to augmented ankle dorsiflexion during landing compared to take-off [20] or discrepancies in the IR beam’s interception during take-off and landing. These variations in the cut-off positions can have an impact on the consistency of measurements.

Therefore, this study aimed to analyze the intrasession reliability of the portable ADR jumping photocell in highly trained female volleyball players and the reliability relative to the emitter when it is located facing the phalanges of the foot (forefoot method) and when it is located facing the metatarsal area (midfoot method). It is hypothesized that the intrasession reliability of ADR jumping will be high in the estimation of the countermovement jump (CMJ) height and that the instrument will be more reliable when a protocol in which the emitter is facing the forefoot is followed.

## 2. Materials and Methods

### 2.1. Participants

A total of 12 highly trained [21] female volleyball players (age 22.8 ± 3.1 years, body mass 63.9 ± 7.5 kg, height 1.72 ± 0.06 m, fat percentage 17.5 ± 3.23, BMI 21.6 ± 2.1 kg/m^2^, experience playing volleyball 9.0 ± 1.9 years) participated voluntarily in this study. Prior to the commencement of the study, all participants were required to provide their informed consent. The informed consent document they signed contained detailed information regarding the characteristics of the intervention, as well as the scientific utilization of the data gathered during the study. The research adhered to the guidelines set forth in the Declaration of Helsinki of The World Medical Association (WMA), Ethical Principles for Medical Research Involving Human Subjects of 1975 (revised in Fortaleza, Brazil in 2013). Moreover, this study was approved by the ethics committee of the University of Alicante (UA-2018-11-17).

To be eligible for the study, all participants were required to meet the following inclusion criteria: be over 18 years of age, possess at least 3 years of experience in playing volleyball, and be familiar with countermovement jumping. Conversely, individuals were excluded from the study if they had a previous musculoskeletal injury or acquired one during the experimental phase, failed to participate in all interventions outlined in the study, or consumed alcohol or drugs within 48 h prior to the testing phase.

### 2.2. Study Design

This was an observational study designed to determine the reliability of jumping tests using a repeated measure design of vertical jumping on the same day, modifying the location of the photocell in relation to the foot between attempts. Data were collected using an ADR jumping photocell in two different configurations, with the IR emitter facing the forefoot and midfoot, to compare the results and determine the instrument’s reliability with respect to emitter location. To achieve a statistical power of 95% at α = 0.05, two-tailed, and an effect size ES > 0.25, G*Power (v3.1.9.7, Heinrich-Heine-Universität Düsseldorf, Düsseldorf, Germany) indicated a minimum of 220 jumps for the Wilcoxon test and 197 jumps for the correlation of two-paired variables. To meet this requirement, the 12 participants executed 20 countermovement jumps in each of the 2 configurations, taking a 2 min rest between each attempt and in each session. This resulted in a total of 240 valid jumps.

### 2.3. Instruments

The CMJ tests were carried out using the ADR jumping photocell, which consists of an IR emitter and receiver measuring 12 × 5 × 2.5 cm that forms an IR barrier on which the feet are placed. The IR beam is emitted by a single red diode (wavelength 940 nm, radiation flux 40 mW), which is received by a dual diode receiver (wavelength 950 nm) that is equipped with a filter to avoid interference from other radiation sources. Jump height, along with other jump-related variables (reactive strength index and contact time) can be read directly as it has a liquid crystal display (LCD) screen on the receiver unit. At the same time, the same data can be simultaneously sent via Bluetooth to a tablet or smartphone using the ADR jumping application that allows the data to be exported. The VJH estimation was carried out by determining the take-off instant (when the IR beam arrives at the receiver) and the landing instant (when the IR beam is cut by the first foot touching the ground). These instants were used to calculate the flight time (*t*) and jump height (VJH) according to the equation *VJH* = *t*^2^ × *g*/8, where *g* represents the acceleration due to gravity (9.8 m/s^2^). In addition to the flight time, the application also provided automated values for contact time, jump height, and the reactive strength index.

### 2.4. Experimental Procedure

The procedure was conducted over two sessions, with a seven-day interval between them, at the sports science laboratory at the University of Alicante. To ensure consistency, all participants underwent the tests at the same time of day and in a room with a temperature of approximately 20 °C. In the first session, anthropometric measurements were taken and familiarization with the experimental protocols was completed. In the second session, data were collected following the following procedure: First, a standardized warm-up was performed consisting of running interspersed with changes of direction during 5 min, followed by 3 min of dynamic stretching and movement exercises. Next, subjects were briefed and practiced on the correct execution of the CMJ during 2 min. After the warm-up, a resting time of 4 min was followed by a verbal review of the protocols. Finally, subjects performed 20 CMJs, alternating between the method that places the transmitter in front of the forefoot and the method that places it on the midfoot, with subjects resting for 2 min between each attempt. Considering that high-level players execute from 65 to 136 jumps per game [22,23,24], the number of CMJs in this procedure were reasonably low to avoid the effects of fatigue [25].

**Figure 1:**
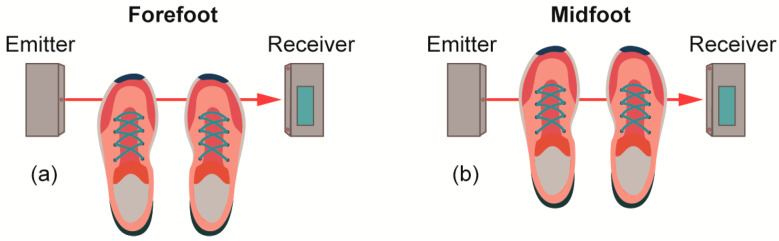
ADR Jumping arrangement with the emitter facing the forefoot (**a**) and midfoot (**b**).

To prevent horizontal and lateral displacements, the jumps were performed over a limited area of 30 *×* 42 cm so that both take-off and landing were executed entirely within these limits. At the lateral ends of the jumping surface, the ADR jumping device was positioned so that the center of the transmitter and receiver boxes were aligned with the phalanges at take-off and landing (forefoot method) or aligned with the metatarsal of the foot (midfoot method), as shown in Figure 1. The forefoot and midfoot positions for each participant were identified and marked within the sports shoe. These markers were subsequently utilized during the execution of jumps to align both feet with the IR beam in accordance with the specified methodology. Furthermore, landing positions were evaluated to confirm proper foot placement.

Participants were instructed to perform CMJs by rapidly descending to a self-selected depth [26,27], followed by a quick ascent to facilitate take-off. During all jumps, participants placed their hands on their iliac crests in the akimbo position [28] to minimize variability caused by arm movement. They were instructed to jump as high as possible and to land on their toes, adopting the ankle joint position during take-off to reduce errors caused by variations in ankle flexion during landing [20]. All jumps were closely supervised by an instructor to ensure proper execution, and attempts were deemed invalid if participants did not land within set limits, did not land on their toes, or removed their hands from their iliac crests during the jump. All trials were sent automatically to a smartphone via Bluetooth and recorded with the ADR jumping app (v. 3.0.0). To verify the data, an instructor annotated each jump height using the information shown in the on-screen display.

### 2.5. Statistical Analysis

The mean and 95% confidence limits were used to present the descriptive data. A Kolmogorov–Smirnov test was used to study normality, revealing that the sample was non-normal. Therefore, Lin’s concordance index (CCC) was calculated to evaluate the level of agreement and presence of systematic error between the two methods. CCC is the product of two factors: *ρ*, precision, providing information on the degree of similarity between the height data obtained through both methods and *C_b_*, accuracy, which quantifies the proximity of the data to the ideal fit [29]. The obtained results are categorized as poor (≤0.9), moderate (0.90–0.95), substantial (0.95–0.99) and near-perfect (≥0.99) [30]. Additionally, a correlation analysis was conducted by computing the Spearman’s bivariate correlation coefficient (*r_s_*), as the non-normal distribution of the sample precludes the utilization of Pearson’s coefficient [31]. The following thresholds were used for the interpretation of *r_s_*: trivial (≤0.1), low (0.1–0.3), moderate (0.3–0.5), high (0.5–0.7), very high (0.7–0.9) and almost perfect (≥0.9) [8,32].

The presence of a linear association between the paired data obtained from both methods was assessed using the Passing and Bablok regression for non-parametric samples [33]. The equation *y* = *ax* + *b* was used to establish the relationship, where the values of a variable *y* can be predicted as a function of another variable *x*. Information on the proportional differences between the two methods is provided by the slope (*a*), which would be equal to one in a perfect pairing. The cut-off point with the *x*-axis representing the systematic difference between the two methods is represented by the intercept (*b*). The calculation of the residual standard deviation from the least square regression or standard error of estimate (SEE) was also carried out. The closeness of the points to the regression line, and, thus, the accuracy of the estimation, was reflected by a lower value of SEE. A Cusum test was performed to evaluate the suitability of Passing and Bablok regression. A small *p*-value (*p* < 0.05) suggests that there is no linear association between the methods (proportional bias).

To investigate the presence of systematic error, the Wilcoxon test for paired samples was employed to determine significant differences in the ADR jumping outcomes obtained from the forefoot and midfoot methods. The effect size (ES) of the differences was determined as Hedges’ corrected effect size *g*, interpreted as trivial (<0.2), small (0.2–0.6), moderate (0.6–1.2), large (1.2–2.0), very large (2.0–4.0), and huge (>4.0) [34].

The degree of agreement between the height data of the two methods was checked using Bland–Altman plots, allowing for the determination of the systematic error and its limits of agreement for 95% (LoA = 1.96 × *SD*). The formula used to compute the maximum permissible differences was based on the coefficients of variation (CV) for each method, which was √(CV^2^_method1_ + CV^2^_method2_) [35]. The methods were considered to be concordant if the 95% confidence intervals of the upper limit of agreement (LoA) fell below the maximum allowable difference and the lower LoA was above the maximum allowable difference [36]. To determine if there were random errors and proportional bias between methods, the bivariate Pearson’s product-moment correlation coefficient of the differences was calculated in the Bland–Altman plots, where an *r*^2^ value greater than 0.1 would indicate their presence.

An estimation of the error magnitude was obtained through the calculation of the standard error of measurement (SEM), which is determined as *S_d_*/√2, where *S_d_* represents the standard deviation of the value differences [37]. This statistical measurement allows for an analysis of the dispersion of values around the true value, providing information on the error in absolute terms [38]. The coefficient of variation (CV) was calculated from SEM as SEM/mean to establish relative reliability. A method is considered reliable if ICC > 0.90 and CV < 5% [7].

The sensitivity of the measurement was evaluated using the smallest worthwhile change (SWC), as the minimum improvement that would have practical significance. To compute SWC as 0.2×√2×SEM, the research question, the type of data, and the available resources in this study were taken into consideration [39,40]. The signal-to-noise ratio (SWC/SEM) was calculated to assess if the method could be considered reliable (SWC/SEM > 1) [41]. The analysis was performed utilizing MedCalc Statistical Software (v 20.100, MedCalc Software Ltd., Ostend, Belgium) and a spreadsheet developed by Hopkins in Sportscience [42].

## 3. Results

Table 1 shows the mean, difference, and the confidence limits for 95% values of the jump height for both methods. Statistically significant differences were observed between the forefoot and midfoot methods, characterized by large effect sizes.

### 3.1. Intrasession Test-Retest Reliability

The intrasession test-retest reliability (consistency) was evaluated using the first five jumps of each method (forefoot and midfoot). To complete this analysis, the pairings of each of the consecutive jumps were analyzed in conjunction with the mean of the test in both methods, as shown in Table 2. The ICC values revealed almost perfect correlations (ICC = 0.95–0.98) for the forefoot and very high correlations (ICC = 0.79–0.93) for the midfoot. On the other hand, the CCC also indicates more reliable values for the forefoot (CCC = 0.95) compared to the lower midfoot values (CCC = 0.81). The analysis of the CCC factors shows that the forefoot results in very high precision values and almost perfect accuracy (*ρ* = 0.94 and *C_b_* = 0.99), higher than the midfoot, in which moderate precision values and almost perfect accuracy are obtained (*ρ* = 0.50 and *C_b_* = 0.92). In the same line, the CVs of the forefoot (4.11%) are lower than those obtained for the midfoot (8.75%).

With regard to the magnitude of errors, SEM values were 1.15 cm (0.96–1.45 cm) and 3.68 cm (2.18–4.70 cm) for the forefoot and midfoot, respectively. Likewise, the sensitivity of the forefoot method was found to be higher than that of the midfoot method, as evidenced by smaller SWC values (0.33 cm) compared to the midfoot method’s values of SWC (1.04 cm). Similarly, the signal-to-noise ratio was less than unity in both cases, which implies that the minimum change with practical interest was greater than the noise.

**Figure 2:**
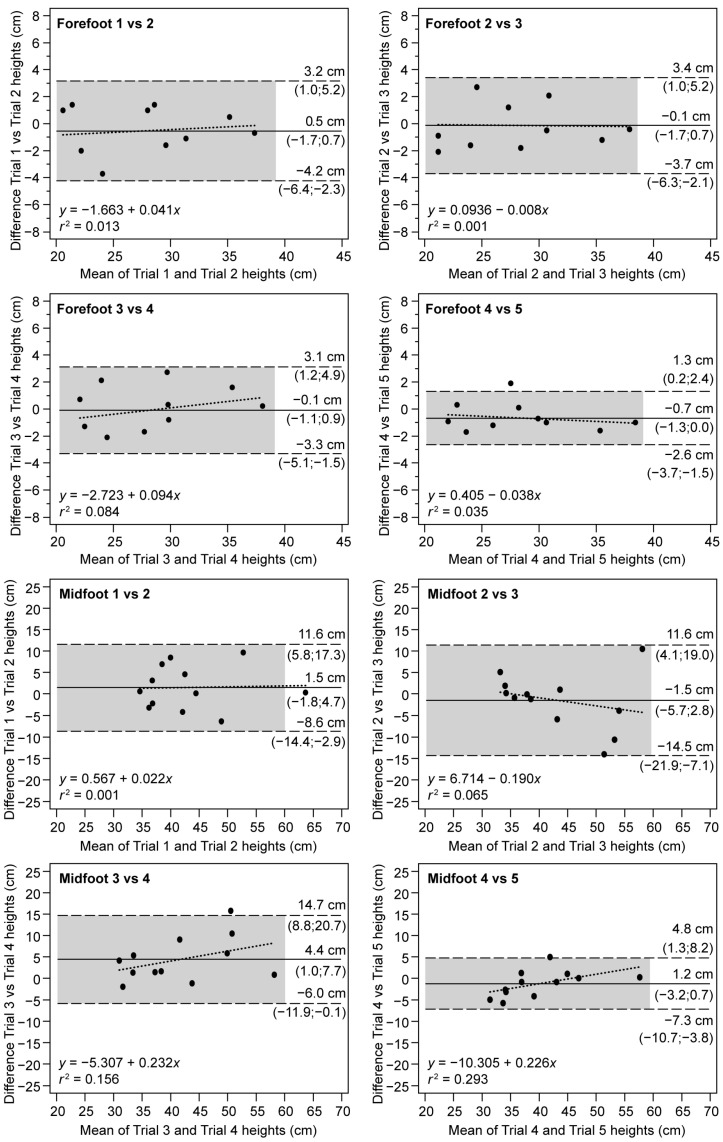
Intrasession reliability for consecutive trials. Bland–Altman plots for consecutive pairs of jumps between trials for forefoot (top), and midfoot (down). Solid line: mean of the differences (systematic error); dashed line: upper and lower LoA (random error); dotted line: regression of the differences (proportional error); shadow area: confidence intervals for 95% of the LoAs and mean.

The consistency between consecutive attempts was analyzed using the Bland–Altman analysis. In Figure 2, a greater dispersion in data for the midfoot method can be observed, which implies greater variability on data collection, although, in both cases, all points are within the upper and lower LoA. The systematic error is lower in the forefoot, with values ranging from −0.7 cm to 0.5 cm for the forefoot and from −1.5 cm to 4.4 cm for the midfoot. On the other hand, the error is not proportional to the measurement range of the forefoot since the Pearson’s correlation is lower than 0.1 (0.001 to 0.048). Greater slopes were found for the midfoot, resulting in lack of homogeneity of variance in the difference between trials (*r*^2^ ranging from 0.001 to 0.293).

### 3.2. Reliability between Methods

Table 3 shows the degree of agreement between the two methods by comparing the jump height data derived from forefoot and midfoot. Statistically significant differences of 13.47 cm were found between the two methods with large ES (*g* = 2.06). The correlation coefficients were moderate for ICC and low for CCC. A random error of SEM = 4.70 cm was obtained with high CVs. The minimum change with practical relevance was 1.33 cm; therefore, the signal-to-noise ratio was below unity (0.66).

**Figure 3:**
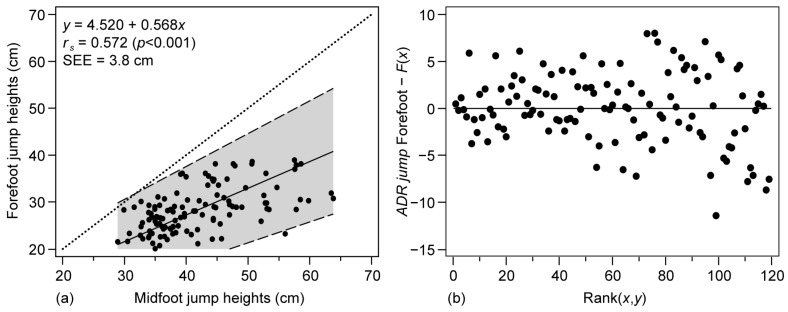
Correlation analysis between the forefoot and midfoot methods in the ADR jumping photocell. (**a**) Passing and Bablok regression, (**b**) residuals plot. Solid line: regression; shaded area: 95% confidence intervals of the regression line; dotted line: *x* = *y* line; *r_s_*: Spearman’s correlation coefficient; SEE = standard error of estimate.

The degree of agreement between the two methods was also estimated from the Passing and Bablok regression plots and the Bland–Altman plot, as shown in Figure 2 and Figure 3, respectively. The Spearman correlation derived from the regression in Figure 2 indicates moderate values (*r_s_* = 0.57, *p* < 0.001). On the other hand, the systematic error between methods can be quantified through the intercept 4.52 cm, and a lack of agreement between methods can be observed through the slope of 0.57 with overestimation in the midfoot method. The SEE of 3.84 cm is a measure of the accuracy of predictions of the fitted line. At the same time, the proportionality of the error is confirmed, as the Cusum test showed significant differences to linearity (*p* < 0.91), a pattern that can be seen visually in the residual graph, with greater dispersion in the larger values.

**Figure 4:**
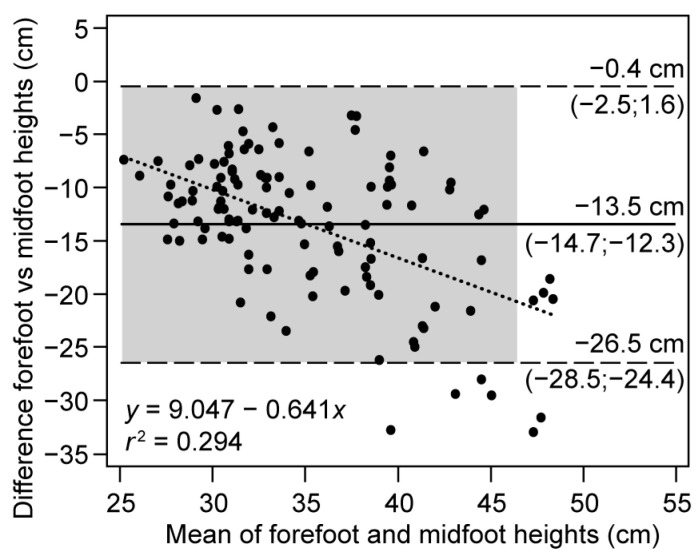
Bland–Altman plot for the study of the reliability of ADR jumping photocell relative to the different locations (forefoot and midfoot) of the IR emitter. Solid line: mean of the differences (systematic error); dashed line: upper and lower LoA (random error); dotted line: regression of the differences (proportional error); shadow area: confidence intervals for 95% of the LoAs and mean.

Figure 4 depicts the Bland–Altman plot between the two methods. Although 95% of the data were included inside the LoAs, representing good agreement, the width of LoAs (−26.5 to −0.4 cm) indicates the presence of random error, all of which lay outside the line of total agreement. The systematic error can be quantified as an overestimation of 13.5 cm for the midfoot method (95% CI: −14.7 to −12.3 cm). In addition, the tendency for the differences to increase in absolute value as the height of the jumps increases indicates proportional error (heteroscedasticity). The negative slope (−0.64, *p* < 0.001) of the fitted line (dotted) shows that the overestimation of the midfoot method increases with jump height (*r^2^* = 0.294).

## 4. Discussion

The purpose of this study was to determine the reliability of the ADR jumping device in two different configurations when the emitter is positioned facing the forefoot and when the emitter is positioned facing the midfoot. To the authors’ knowledge, this study is the first to analyze the reliability relative to the cut-off position of the infrared beam of the photocell with respect to the foot. This topic is of interest since the reliability of the device may be compromised as a function of measurement configuration. The main result of this research showed that the ADR jumping device is a reliable instrument for measuring vertical jumping when the device is positioned so that the emitting beam faces the forefoot at take-off and landing. In addition, the positioning of the foot with respect to the emitter affects the reliability of the results.

The consistency of the ADR jumping device was analyzed by determining the intrasession reliability. Our results showed high consistency of measurement for the ADR jumping photocell, with near-perfect ICC values for the forefoot and high CCC between consecutive pairs, considering near-perfect correlations in all pairings (ICC: 0.95–0.98, CCC: 0.93–0.97) and random error of SEM = 1.15 cm. However, the results of the midfoot method were lower, as shown by ICC and CCC, representing very high and poor correlations, respectively. In addition, SEM values tripled (3.68 cm). Similarly, higher CV was found for rearfoot (4.1%) than for midfoot (8.7%).

The moderate consistency found for the midfoot contrasted with the high levels of consistency observed for the forefoot, which are comparable to those reported in other studies. Near-perfect ICCs were found for Optojump (ICC = 0.98; CV = 2.2%) and SEM = 2.43 cm [15], Wheeler jump (CV = 1.5%) and SEM = 1.33 cm [19], Fitjump (ICC = 0.97; CV = 2.3%) and SEM = 1.30 cm [17], and ADR jumping itself (ICC = 0.89, SEM = 0.7 cm) [18]. However, consistency was examined in these studies from sessions separated by 7 days (intersession reliability), which may explain the lower ICC reported in [18], as variability may increase due to biological or environmental factors. In a prior investigation on the validity of ADR jumping, it was shown that ADR jumping was valid when compared to a motion capture system. Additionally, it was deemed reliable in a within-session test (ICC = 0.96; SEM = 1.18 cm; CV = 4.21%), when measurements were taken at the forefoot and derived from the initial five jumps of a single session [43].

Additionally, the forefoot method exhibited a greater sensitivity in detecting ADR jumping, with an SWC of 0.32 cm. Thus, the noise (SEM) is greater than the minimum change of practical interest, so the validity of this device may be questionable in populations where slight variations have a significant impact on performance, such as in a group of elite athletes. On the other hand, the midfoot sensitivity is lower, with an SWC value that is almost three times those found for the midfoot. These results showed that the beam cut-off zone is a relevant factor affecting the sensitivity of the instrument, with the forefoot zone being more reliable.

All observations, thus far, suggest that there are variations in reliability between the midfoot and forefoot methods. In fact, statistically significant differences were observed with a large effect size for the paired differences between both methods. This implies an overestimation of 13.5 cm for the midfoot method compared to the forefoot method, which is quite substantial, representing between 30% and 48% of the means of the forefoot and midfoot methods, respectively. Furthermore, the correlation coefficients between the two methods were low, with an ICC = 0.49 (moderate) and a CCC = 0.15 (poor). Likewise, the high levels of noise, probably generated by the scattered data, mean that the sensitivity to detect changes between both methods yields an SWC of 1.3 cm, implying that the minimum practical change is less than the noise generated.

The regression analysis showed low correlations (*r_s_* = 0.45) with systematic errors of 4.5 cm (intercept) and relative errors of 0.57 (slope), as well as a random error of 3.8 cm (SEE), thus showing a lack of association between devices. Furthermore, the Bland–Altman plots confirmed these trends: the overestimation of the midfoot over the forefoot was 13.5 cm, and the observed data dispersions were large, which can be visually noticed by the separation of 26 cm between the upper and lower LoA. Likewise, a high proportional error (slope −0.64) was observed because, as values increase, the spread of the data becomes greater, resulting in larger differences between methods. Finally, the entirety of the LoA fell outside the range of acceptable differences, indicating poor intermethod reliability.

To the best of the author’s knowledge, no studies have compared the reliability of the location of the cut-off point of the emitted beam in photocells. However, according to our results, the foot cut-off zone can have a significant influence on the measured jump height. The technical information of the manufacturer indicates two valid positions for the location of the photocell, forefoot, and midfoot. However, based on the findings of this study, these locations are not interchangeable. The key aspect for flight time-based systems is the accurate detection of the take-off and landing phases. In photocells, these moments are determined when the beam is no longer interrupted by the foot. Therefore, positioning the cut-off zone in the forefoot implies that it is cut off when ankle extension is complete, thus the data obtained can be considered interchangeable with data from jump mats [18,19] or force platforms [44]. On the other hand, if the device is positioned so that the beam is placed at the midfoot, the flight time will increase because the emitted beam picks up the signal of the first cut by default. The use of this method may result in an overestimation of jump height due to the cut-off point of the beam being recorded at the heel rather than the midfoot. This would cause a delay in the recording of the take-off and landing phases, leading to an overestimation of the measurement, thereby compromising the validity of the instrument. Furthermore, the midfoot method also has the potential to affect the reliability of the instrument, as the delayed cut-off point can cause systematic error due to an elongated flight time being recorded. Additionally, recording data from the heel can result in random error, due to the greater variability caused by the flexion of the ankle and knee, as it does not capture the maximum joint extension point (landing and take-off) but rather an intermediate and nonstandardized point. The combination of these factors, along with already known sources of variability such as biological variation, horizontal or lateral displacements during the flight phase, and the cushioning effect of cushioned surfaces or footwear [15,16], can greatly decrease the reliability of the device by delaying the beam cut-off point.

One limitation of our study is the small sample size, which consisted of twelve highly trained female volleyball players. While this sample was selected based on their expertise and experience in the field, the small sample size may limit the generalizability of our findings to other populations. We acknowledge that a larger sample size may have improved the statistical power of our study and reduced the potential for type II errors. However, given the specificity of the population and the difficulty in recruiting highly trained female volleyball players, we believe that the sample size used in this study is appropriate.

## 5. Conclusions

The ADR jumping photocell was found to be a consistent tool for measuring the vertical jump, with high levels of reliability in the intrasession test. However, the positioning of the emitter can have a significant impact on the reliability and sensitivity of the instrument. Specifically, ADR jumping is a reliable instrument for measuring vertical jumping when the device is positioned so that the emitting beam faces the forefoot at take-off and landing, but the reliability of the midfoot method was lower. Although the manufacturer’s technical information indicates two valid positions for the location of the photocell, forefoot and midfoot, our findings suggest that these positions are not interchangeable.

The findings of this study emphasize the significance of proper emitter positioning in achieving precise measurements. These results could have significant implications for researchers, coaches, and athletes who rely on ADR jumping devices to measure vertical jump performance.

## Figures and Tables

**Table 1:** Mean and 95% CI jump height derived from forefoot and midfoot methods.

	Forefoot	Midfoot	Difference	*p*	ES (*g*)
Jump height (cm)	28.39	41.86	−13.47 *	*p* < 0.001	2.1 (large)
95% CI	27.54 to 29.26	40.42 to 43.30	−14.67 to −12.26	-	1.7 to 2.4

* Statistically significant difference, CI = confidence interval, ES = Hedges’ corrected effect size.

**Table 2:** Intrasession test-retest reliability for the ADR jumping photocell located in the forefoot and midfoot cutting beam.

	Forefoot					Midfoot				
	2–1	3–2	4–3	5–4	Mean	2–1	3–2	4–3	5–4	Mean
Change in mean	0.54	0.13	0.10	0.67	-	−1.48	1.46	−4.37	1.24	-
CI-95% lower	−0.44	−0.81	−0.75	0.15	-	−4.16	−1.99	−7.11	−0.36	-
CI-95% upper	1.52	1.08	0.95	1.19	-	1.19	4.91	−1.62	2.84	-
ICC	0.95	0.95	0.96	0.98	0.96	0.86	0.79	0.87	0.93	0.85
CI-95% lower	0.87	0.87	0.89	0.96	0.92	0.65	0.51	0.67	0.82	0.72
CI-95% upper	0.98	0.98	0.99	0.99	0.99	0.95	0.92	0.95	0.98	0.94
CCC	0.93	0.94	0.95	0.97	0.95	0.86	0.75	0.75	0.90	0.81
CI-95% lower	0.79	0.81	0.84	0.91	0.83	0.49	0.36	0.42	0.74	0.50
CI-95% upper	0.99	0.98	0.98	0.99	0.98	0.94	0.91	0.90	0.96	0.92
*ρ* (precision)	0.94	0.94	0.95	0.98	0.95	0.82	0.77	0.86	0.94	0.85
*C_b_* (accuracy)	0.99	0.99	0.99	0.99	0.99	0.98	0.97	0.87	0.96	0.94
SEM (cm)	1.34	1.28	1.16	0.71	1.15	3.65	4.70	3.74	2.18	3.68
CI-95% lower	1.00	0.96	0.86	0.53	0.96	2.73	3.52	2.80	1.63	3.09
CI-95% upper	2.07	1.99	1.79	1.10	1.45	5.65	7.29	5.81	3.39	4.63
CV (%)	4.90	4.62	4.14	2.52	4.11	8.32	11.11	8.55	5.54	8.75
SWC (cm)	0.38	0.36	0.33	0.20	0.32	1.03	1.33	1.06	0.62	1.04
CI-95% lower	0.28	0.27	0.24	0.15	0.27	0.77	0.99	0.79	0.46	0.88
CI-95% upper	0.59	0.56	0.51	0.31	0.41	1.60	2.06	1.64	0.96	1.31

CI = 95% confidence intervals; ES = effect size; ICC = intraclass correlation coefficient; CCC = Lin’s coefficient of agreement; *ρ* = precision derived from CCC; Cb = accuracy derived from CCC; SEM = standard error of the measurement; CV = coefficient of variation; SWC = minimum worthwhile change.

**Table 3:** Between method reliability for the ADR jumping photocell.

	Forefoot vs. Midfoot	95% CI
Mean difference (cm)	13.47 *	12.46–14.48
ES (*g*)	2.06	1.7–2.4
ICC	0.49	0.36–0.59
CCC	0.15	0.10–0.21
*ρ* (precision)	0.55	-
*C_b_* (accuracy)	0.28	-
SEM (cm)	4.70	4.25–5.27
SEM_std_	0.72	0.65–0.81
CV (forefoot) (%)	16.7	-
CV (midfoot) (%)	18.9	-
SWC (cm)	1.33	1.20–1.49
SNR	0.66	

95% CI = 95% confidence intervals; ES = effect size; ICC = intraclass correlation coefficient; CCC = Lin’s coefficient of agreement; *ρ* = precision and *C_b_* = accuracy derived from CCC; SEM = standard error of the measurement; CV = coefficient of variation; SWC = minimum change with practical impact; SNR = signal-to-noise ratio. * Statistically significant differences (*p* < 0.001).

## Data Availability

The data presented in this study are available on reasonable request from the corresponding author.
